# Socioeconomic disadvantage contributes to ethnic disparities in multiple myeloma survival: a matched cohort study

**DOI:** 10.1038/s41408-022-00681-x

**Published:** 2022-05-25

**Authors:** Christopher Staffi Buradagunta, Zhuping Garacci, Anita D’Souza, Binod Dhakal, Sumana Devata, Siegfried Janz, Aaron P. Thrift, Parameswaran Hari, Melinda Stolley, Jing Dong

**Affiliations:** 1 Division of Hematology Oncology, Department of Medicine, Medical College of Wisconsin, Milwaukee, WI USA; 2 Center for Advancing Population Science, Medical College of Wisconsin, Milwaukee, WI USA; 3 Center for International Blood and Marrow Transplant Research, Medical College of Wisconsin, Milwaukee, WI USA; 4 Section of Hematology Oncology, Department of Medicine, Clement J Zablocki Veterans Affairs Medical Center, Milwaukee, WI USA; 5 Medical College of Wisconsin Cancer Center, Milwaukee, WI USA; 6 Section of Epidemiology and Population Sciences, Department of Medicine, and Dan L Duncan Comprehensive Cancer Center, Baylor College of Medicine, Houston, TX USA

**Keywords:** Risk factors, Myeloma

**Dear Editor**,

Multiple myeloma (MM) is the second most common hematologic malignancy in the US [1]. Despite therapeutic advances and overall improved survival, large racial and ethnic disparities in MM survival still exist [2, 3]. Studies using registry and trial data suggest that racial/ethnic minority patients, such as Hispanics and non-Hispanic blacks (NHBs), are disproportionately affected by poor socioeconomic status (SES) and have a lower utilization rate of novel therapeutic agents (e.g., proteasome inhibitors [PIs] and immunomodulatory drugs [IMiDs]) and autologous stem cell transplantation (ASCT) than non-Hispanic white (NHW) patients [2, 4]. However, whether this translates into inferior outcomes remains inconclusive [2, 3, 5–8]. Previous studies applied model-based methods which, when fitted to the entire population, give disproportionate weighting to the majority population (i.e., NHW) [9]. Using a tapered matching approach, we recently investigated the Surveillance, Epidemiology and End Results (SEER) -Medicare linked database to examine racial disparities in MM survival and associated factors in 3319 NHB and 20,831 NHW patients [10]. Consistent with previous reports [3, 5, 11, 12], we found NHBs have a significantly longer overall survival than NHWs when treated similarly [10]. However, data on Hispanics, the fastest growing segment of the US population, is limited. Herein, we applied the matching approach on the same SEER-Medicare database to examine the sequential effects of demographics, clinical, and treatment-related factors on survival disparities between Hispanic and NHW patients with MM.

This study was approved by the Institutional Review Board at the Medical College of Wisconsin. We identified 1591 Hispanic and 20,831 NHW patients, 65 years or older, diagnosed with MM between 1999 and 2017 and followed up through 2018. Four sets of 1591 NHW patients were matched sequentially, using propensity score matching approach [9, 10, 13–15], to the same set of 1591 Hispanic patients based on demographics (age, sex, year of diagnosis, SEER site, and marital status), SES (demographic variables plus SES), presentation (SES variables plus comorbidities) and treatment (presentation variables plus traditional chemotherapy, PIs, IMiDs and ASCT). Details in patient selection and matching process are described elsewhere [10].

Compared to the unmatched NHWs, Hispanics were younger on average (75.8 vs. 77.1 years), more likely to be female (52.6% vs. 48.0%), to be unmarried at diagnosis (30.6% vs. 25.2%), and to have low SES (52.6% vs. 23.1%). Hispanics also had more comorbidities (Comorbidity Index ≥1, 83.8% vs. 78.0%), were more likely to receive IMiDs (23.6% vs. 19.7%), but less likely to receive ASCT (4.0% vs. 5.3%) than NHWs (all *P* < 0.05, Table 1).

**Table 1:** Characteristics of hispanic and non-hispanic white patients.

Variable	Hispanic patients (*n* = 1591)	Non-Hispanic white patients, *n* (%)				
		Treatment-matched (*n* = 1591)	Presentation- matched (*n* = 1591)	SES- matched (*n* = 1591)	Demographics- matched (*n* = 1591)	All Whites- unmatched (*n* = 20,831)
Mean diagnosis year (SD)	2008.9 (5.06)	2008.7 (5.12)	2009.0 (5.02)	2008.6 (4.97)	2008.8 (4.86)	**2008.4 (5.14)**
Mean age at diagnosis (SD), y	75.8 (6.79)	75.9 (6.74)	75.9 (6.70)	75.8 (6.60)	75.8 (6.78)	**77.1 (6.93)**
Female	837 (52.61)	829 (52.11)	798 (50.16)	823 (51.73)	837 (52.61)	**9992 (47.97)**
Marital status						
Married	579 (36.39)	585 (36.77)	575 (36.14)	562 (35.32)	579 (36.39)	**8376 (40.21)**
Not married	486 (30.55)	494 (31.05)	479 (30.11)	501 (31.49)	486 (30.55)	**5255 (25.23)**
Unknown	526 (33.06)	512 (32.18)	537 (33.75)	528 (33.19)	526 (33.06)	**7200 (34.56)**
SES						
Low	836 (52.55)	855 (53.74)	852 (53.55)	836 (52.55)	**321 (20.18)**	**4809 (23.09)**
Moderate	510 (32.06)	527 (33.12)	493 (30.99)	510 (32.06)	**770 (48.40)**	**10,062 (48.30)**
High	245 (15.40)	209 (13.14)	246 (15.46)	245 (15.40)	**500 (31.43)**	**5960 (28.61)**
Charlson comorbidity score						
0	257 (16.15)	245 (15.40)	257 (16.15)	**352 (22.12)**	**352 (22.12)**	**4576 (21.97)**
1–2	535 (33.63)	567 (35.64)	535 (33.63)	**600 (37.71)**	**650 (40.85)**	**7738 (37.15)**
> = 3	799 (50.22)	779 (48.96)	799 (50.22)	**639 (40.16)**	**589 (37.02)**	**8517 (40.89)**
Chemotherapy						
No	1383 (86.93)	1383 (86.93)	1403 (88.18)	1403 (88.18)	1403 (88.18)	**18,616 (89.37)**
Yes	208 (13.07)	208 (13.07)	188 (11.82)	188 (11.82)	188 (11.82)	**2215 (10.63)**
PIs						
No	1059 (66.56)	1059 (66.56)	1026 (64.49)	1060 (66.62)	1032 (64.86)	14,142 (67.89)
Yes	532 (33.44)	532 (33.44)	565 (35.51)	531 (33.38)	559 (35.14)	6689 (32.11)
IMiDs						
No	1216 (76.43)	1216 (76.43)	1248 (78.44)	1236 (77.69)	1242 (78.06)	**16,722 (80.27)**
Yes	375 (23.57)	375 (23.57)	343 (21.56)	355 (22.31)	349 (21.94)	**4109 (19.73)**
ASCT						
No	1528 (96.04)	1528 (96.04)	1518 (95.41)	1513 (95.10)	**1493 (93.84)**	**19,726 (94.70)**
Yes	63 (3.96)	63 (3.96)	73 (4.59)	78 (4.90)	**98 (6.16)**	**1105 (5.30)**

Note: The samples in the columns are non-overlapping (or minimally overlapping) samples and do not violate SEER-Medicare cell size suppression rule. Variables controlled in some of the 4 matches but allowed to vary naturally in other matches. The “Hispanic patients” column reports the statistical numbers for all Hispanic patients in the data set. The “Treatment-matched” column reports the statistical numbers for the closest non-Hispanic white match, namely the treatment match (which also controls for presentation, SES, and demographic variables); the “Presentation-matched” column also controls for SES and demographic variables; the “SES-matched” column also controls for demographic variables. The “All Whites-unmatched” column reports data for all non-Hispanic whites in the data set without matching. Results for each variable that appear to the left of the bold vertical line are for variables included in the match designated by the column. Results to the right of the bold vertical line are for variables not used in the match designated by the column. Percentages or rates bolded imply statistically significant (*P* < 0.05) differences between Hispanics and non-Hispanic whites.

Overall, 1217 of 1591 Hispanics (76.5%) and 16,479 of 20,831 NHWs (79.1%) died. Compared with demographics matched NHWs, Hispanics had a significantly shorter median survival (30.0 vs. 37.0 months; *P* = 0.004). However, after matching for SES, the difference in median survival was no longer significant (30.0 vs. 32.0 months, *P* = 0.46), neither in the presentation match (30.0 vs. 28.0 months, *P* = 0.38) nor in the treatment match (30.0 vs. 29.0 months, *P* = 0.19). Likewise, 5-year survival rates differed between Hispanics and demographics-matched NHWs (absolute 5-year survival difference, 3.6%*, P* = 0.002), but after matching for SES, this difference in 5-year survival was reduced to 2.2% and was not statistically significant (*P* = 0.32). No 5-year survival difference was observed in the presentation or treatment match (both *P* > 0.10, Fig. 1A). The results from the Cox regression analysis mirrored those of the matching approach. Hispanic ethnicity was significantly associated with increased mortality risk in models adjusted for demographics variables (Hispanic vs. NHW: hazard ratios [HR], 1.16; 95% confidence interval [CI], 1.05–1.30, *P* = 0.005); however, Hispanic ethnicity was not associated with mortality after additionally adjusting for SES, presentation, or treatment factors (all *P* > 0.10). These results suggest that SES accounted for the survival disparities between Hispanic and NHW patients with MM. We further conducted stratified analyses by SES to identify factors contributing to the survival disparity for patients with similar SES. We found if Hispanics and NHWs were both at high SES, they experienced similar survival across the demographics, presentation, and treatment match (all *P* > 0.10). However, among those with low SES, Hispanics still had 18% excess risk of all-cause mortality compared to demographics matched NHWs (95% CI, 1.02–1.37, *P* = 0.027). Further matching on presentation and treatment eliminated these survival differences (all *P* > 0.10), suggesting the important role of comorbidities and treatment factors in MM survival disparities among low-SES patients.

**Fig. 1:**
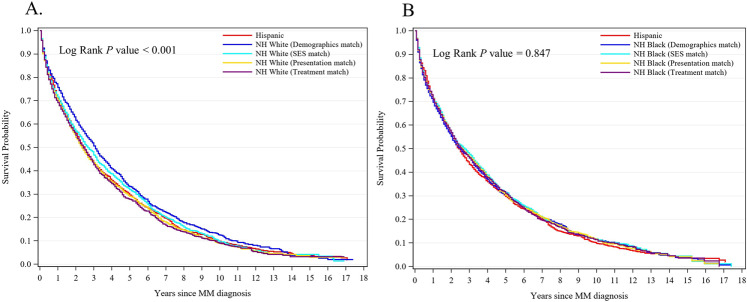
Survival curve. **A** Life-Table plot for multiple myeloma survival for the total Hispanic population (*n* = 1591) and the 4 matched non-Hispanic white populations (each *n* = 1591) diagnosed between 1999 and 2017. **B** Life-Table plot for multiple myeloma survival for a subset Hispanic population (*n* = 1548) and the 4 matched non-Hispanic black populations (each *n* = 1548) diagnosed between 1999 and 2017.

Intriguingly, although Hispanics and NHBs are both underserved populations, we discovered different survival outcomes compared to NHWs using the same database and matching approach [10]. To elucidate the underlying reasons for these survival differences, we conducted a subcohort analysis in 1548 Hispanics who had 4 sets of successfully matched NHBs. Compared to the unmatched NHBs (*n* = 3319), Hispanics were more likely to be married at diagnosis (36.1% vs. 28.3%), to have high SES (14.9% vs. 9.8%), but fewer comorbidities (Comorbidity Index ≥3, 50.3% vs. 53.6%). Hispanics were also more likely to receive chemotherapy (12.7% vs. 9.6%), IMiDs (22.4% vs. 16.6%), and PIs (32.8% vs. 28.2%) than NHBs. The disparities in receipt of IMiDs and PIs persisted even after matching on SES and presentation factors (IMiDs: 22.4% vs. 16.9%, PIs: 32.8% vs. 29.4%, respectively, all *P* < 0.05). These patterns of differences were similar as we observed in the comparisons between NHWs and NHBs [10]. The 5-year survival was similar between Hispanics and the 4 sets of matched NHBs, at 29.7% among Hispanics, 30.3% among demographics matched NHBs, and 31.5%, 30.2%, 31.1% among SES-, presentation-, and treatment-matched NHBs, respectively (all *P* > 0.1, Fig. 1B). When stratified by SES, findings were similar to comparisons of Hispanics and NHWs. Hispanics and NHBs experienced comparable survival across the demographics, presentation, and treatment match (all *P* > 0.1) within high SES strata. However, low-SES Hispanics had a significantly shorter 5-year survival than low-SES NHBs in the demographics match (25.6% vs. 29.4%, *P* = 0.045). These differences were abolished after matching on presentation and treatment factors (both *P* > 0.1), again highlighting the critical role of comorbidities and treatments.

Our study showed that among Medicare beneficiaries, Hispanics had a higher utilization rate of IMiDs and lower utilization rate of ASCT than NHWs. This is consistent with previous reports [4, 16]. More importantly, we observed striking differences in SES between Hispanics and NHWs with MM, which was a highly suggestive contributor to the survival disparity. Stratification analysis showed this survival disparity was only significant among demographics matched pairs with low SES, which was eliminated after further matching on comorbidities and treatment factors. These results highlight the need to better understand factors beyond SES that impact MM survival disparities. However, limited by the large number of missing values in disease-specific mortality, we were not able to assess MM-specific mortality to further examine whether comorbidities and treatments also impact MM-specific mortality. Of note, both comorbidities and treatment are important modifiable factors that may be affected by SES, providing us opportunities to eliminate these disparities. Even though Hispanics and NHBs demonstrate similar and greater social and health challenges than NHWs at diagnosis, the survival disparities between these two underserved populations and NHWs are markedly different. When treated similarly, Hispanics and NHWs have comparable overall survival while NHBs have significantly longer survival than NHWs [10]. In addition, among patients with low SES, NHBs have better survival than demographics-matched Hispanics. These findings indicate NHBs may harbor a more indolent disease subtype than other racial/ethnic groups [17]. Studies have reported that NHBs have significantly lower frequency of “high-risk” MM cytogenetic abnormalities *t*(4;14) and del(17/17p) than NHWs [18]. However, due to the lack of data, we were not able to investigate myeloma cytogenetic risk—a known MM prognostic factor. Further study of disease biology among Hispanic MM patients is needed. In addition, we focused on individual therapeutic class in the current analysis. We cannot rule out the influence of combined treatments on survival outcomes, such as PI plus IMiD triplet combination versus given individually as a doublet. Future research should explore additional social, clinical, and biological factors to understand the mechanisms underlying survival disparity in patients with low SES, so proper intervention and policy development can be implemented.

## Supplementary information

checklist

## Data Availability

The data presented in this study are available publicly on the Surveillance, Epidemiology, and End Results Program (SEER) website and database (https://seer.cancer.gov).
